# Accuracy of a pediatric early warning score in the recognition of clinical deterioration^1^

**DOI:** 10.1590/1518-8345.1733.2912

**Published:** 2017-07-10

**Authors:** Juliana de Oliveira Freitas Miranda, Climene Laura de Camargo, Carlito Lopes Nascimento, Daniel Sales Portela, Alan Monaghan

**Affiliations:** 2 PhD, Assistant Professor, Departamento de Saúde, Universidade Estadual de Feira de Santana, Feira de Santana, BA, Brazil.; 3 PhD, Associate Professor, Escola de Enfermagem, Universidade Federal da Bahia, Salvador, BA, Brazil.; 4 PhD, Full Professor, Departamento de Saúde, Universidade Estadual de Feira de Santana, Feira de Santana, Bahia, Brazil.; 5 MSc, Assistant Professor, Centro de Ciências da Saúde, Universidade Federal do Recôncavo da Bahia, Santo Antônio de Jesus, BA, Brazil.; 6 MSc, Senior Lecturer, University of Brighton, Brigthon, England, United Kingdom.

**Keywords:** Alert, Signs and Symptoms, Child, Hospitalized, Pediatric Nursing, Validation Studies

## Abstract

**Objective:**

to evaluate the accuracy of the version of the Brighton Pediatric Early Warning Score translated and adapted for the Brazilian context, in the recognition of clinical deterioration.

**Method:**

a diagnostic test study to measure the accuracy of the Brighton Pediatric Early Warning Score for the Brazilian context, in relation to a reference standard. The sample consisted of 271 children, aged 0 to 10 years, blindly evaluated by a nurse and a physician, specialists in pediatrics, with interval of 5 to 10 minutes between the evaluations, for the application of the Brighton Pediatric Early Warning Score for the Brazilian context and of the reference standard. The data were processed and analyzed using the Statistical Package for the Social Sciences and VassarStats.net programs. The performance of the Brighton Pediatric Early Warning Score for the Brazilian context was evaluated through the indicators of sensitivity, specificity, predictive values, area under the ROC curve, likelihood ratios and post-test probability.

**Results:**

the Brighton Pediatric Early Warning Score for the Brazilian context showed sensitivity of 73.9%, specificity of 95.5%, positive predictive value of 73.3%, negative predictive value of 94.7%, area under Receiver Operating Characteristic Curve of 91.9% and the positive post-test probability was 80%.

**Conclusion:**

the Brighton Pediatric Early Warning Score for the Brazilian context, presented good performance, considered valid for the recognition of clinical deterioration warning signs of the children studied.

## Introduction

The hospital should be considered a safe place for the prompt care of patients with clinical deterioration; however, the late recognition and treatment of these patients in the hospital environment has been evidenced^1^. The greater complexity of patients admitted to the wards, the difficulties of some professionals in recognizing the severity, and the shortage of trained urgency and emergency staff are examples of conditions that may lead to delays in the recognition of clinical deterioration in hospitalized children^2^ ^-^ ^5^.

Considering this scenario, since 2005, discussions in the literature regarding the need to develop instruments capable of indicating early the risk of clinical deterioration in hospitalized children have been expanded, considering that these tools already exist in the hospital spaces for adult patients, known as Early Warning Scores (EWS)^6^ ^-^ ^9^.

In the pediatric context, the EWS were named Pediatric Early Warning Scores (PEWS), translated into Portuguese as “*escores pediátricos de alerta precoce*”. The first published PEWS was the Brighton Pediatric Early Warning Score (BPEWS), in 2005^6^, and some of its versions have been adapted/modified and validated in specific studies^10^ ^-^ ^12^. The final score of this instrument can vary from 0 to 13 points, obtained from partial scores, based on clinical criteria, organized into three components (neurological, cardiovascular and respiratory), as well as the need for nebulization and the occurrence of post-surgical vomiting^6^.

The BPEWS has been translated and adapted to the Brazilian context (BPEWS-Br)^13^, however, its accuracy in identifying signs of clinical deterioration in hospitalized children has not been tested, which makes it difficult to adopt it in the clinical practice, since validity is an essential property for the use of health measurement instruments.

Therefore, the aim of this study was to evaluate the accuracy of the version of the Brighton Pediatric Early Warning Score translated and adapted for the Brazilian context (BPEWS-Br) in the recognition of clinical deterioration.

## Method

This was a diagnostic test study to verify the accuracy of the BPEWS-Br in the recognition of warning signs of clinical deterioration in hospitalized children, when compared to a reference standard. To guide the method, the Quality Assessment of Diagnostic Accuracy Studies (QUADAS) was used, this being a tool that evaluates the quality of diagnostic accuracy studies^14^.

The accuracy or validity of a diagnostic test refers to its usefulness in diagnosing or predicting a particular event. To verify the validity of a test, its measurement must be made in relation to a gold standard or reference standard^15^.

### Reference standard and cut-off point of the BPEWS-Br for clinical deterioration

Diagnostic test studies need a gold/reference standard that establishes the presence or absence of a disease/event. When it is not possible to determine a gold standard, clinical criteria based on the history and physical examination can be used to establish a diagnosis^16^.

In studies that validate pediatric early warning scores, certain authors have reported difficulty in establishing a reference standard for clinical deterioration in children^8^ ^,^ ^10^ ^,^ ^17^. Some of these have used the call for the Rapid Response Team (RRT)^11^, while others have adopted the transfer to the Intensive Care Unit (ICU), however, they recommended that more standards should be tested^10^ ^,^ ^18^.

In this study, considering that a PEWS aims for the early identification of signs of clinical deterioration; that there is no consensus reference standard for this event; that there is a shortage of pediatric ICU beds in the municipality and a lack of an RRT in the study scenario, the classification of children “without signs of deterioration” and “with signs of deterioration” was made guided by a set of criteria based on the Primary Clinical Evaluation of the Severely Ill Child, recommended by the American Heart Association (AHA) and the American Academy of Pediatrics (AAP)^19^.

Among the criteria of the Primary Clinical Evaluation of the Severely Ill Child, blood pressure was excluded, because it was a late sign of cardiovascular decompensation in the child, as were the Glasgow Coma Scale and the pupillary reaction, opting for the use of the AVPU Pediatric Response Scale (Alert, Responds to voice, Responds to pain and Unresponsive) for rapid neurological assessment^19^.

From a broad discussion among the researchers of this study regarding the reference standard adopted, it was defined that 3 or more altered clinical signs in the primary clinical evaluation would classify the child as “with signs of deterioration”.

Regarding the BPEWS-Br, the score to trigger deterioration was defined by the best cut-off point obtained by the ROC curve. The BPEWS-Br ≥3 was able to maximize sensitivity and specificity and obtained excellent accuracy. Thus, children with a final score <2 were considered “without warning” and those ≥3 “with warning signs for clinical deterioration”.

### Scenario and sample

The scenarios were the units of clinical-surgical hospitalization and observation/stabilization of the emergency sector of a pediatric reference hospital with 280 beds in the city of Feira de Santana. The municipality has approximately 617 thousand inhabitants and is located in the state of Bahia, Brazil.

Inclusion criteria were children from 0 to 10 years of age, hospitalized in the units, regardless of length of hospitalization. Although the original instrument was developed for use with children and adolescents, it was decided to only include children, since this is the population most attended in the units studied. Exclusion criteria were children with medical discharge prescribed, hospitalized in the cardiology or oncology units and those with precautionary measures. Children with heart disease were excluded because there is already a validated warning score for this population in the literature^20^. Oncology children were excluded due to low immunity restricting their exposure and children with precautionary measures because of the risk of cross infection during the collection.

The sample consisted of 271 children from 0 to 10 years of age, hospitalized between May and October 2015, in these units (108 children in clinical medical, 54 in clinical surgery, 30 in nephrology, 65 in observation and 14 in stabilization). Due to the absence of national data on the prevalence of clinical deterioration in hospitalized children, the sample calculation was performed by applying a pilot test with 30 children, for verification, using the reference standard adopted. The estimated value of the expected proportion of children with clinical deterioration used in the sample calculation was 20%.

For each day of data collection, one unit was drawn, and the children admitted to that unit, who fulfilled the inclusion criteria and ethical criteria, participated in the study regardless of whether or not they showed signs of clinical deterioration, considering that in diagnostic test studies it is necessary to have sick and healthy patients.

### Data collection

Three instruments were used in the collection: sociodemographic and clinical identification variables of the children and their families, the reference standard for clinical deterioration and the version of the BPEWS translated and adapted for the Brazilian context (BPEWS-Br).

A pediatrician was trained in the application of the reference standard and a pediatric nurse was trained in the application of the BPEWS-Br. For the theoretical training, an operational manual constructed to guide the measurement of clinical indicators was read and discussed. For the practical training, sessions were performed with videos and clinical cases. After this phase, the pilot test was applied with 30 children.

After the pilot test, the sample was calculated and the data were collected. The evaluations of the children by the physician and the nurse were performed blindly, so that one did not know of the evaluation of the other, at intervals of 5 to 10 minutes, to avoid considerable changes in the clinical condition of the patients.

### Data analysis

Two databases were constructed in EpiData 3.1 to organize the information and identify possible data entry errors. The Statistical Package for the Social Sciences (SPSS^®^), version 9.0 for Windows, and VassarStats.net were used to analyze the data.

For the qualitative variables, simple, absolute and relative frequencies were calculated. In order to test the validity of the BPEWS-Br, compared to the reference standard, the prevalence of clinical deterioration estimated by the reference standard and by the test, the sensitivity, the specificity, the Receiver Operating Characteristic Curve (ROC curve) and the area under the ROC curve, the Positive Predictive Value (PPV), the Negative Predictive Value (NPV), the Positive Likelihood Ratio (LR+), the Negative Likelihood Ratio (LR-) and post-test probability were calculated^21^.

The pre-test probability, required to verify the post-test probability, corresponded to the proportion of clinical deterioration in the pilot test (20%), since the pre-test probability of clinical deterioration in the pediatric population was unknown. Data were presented in the form of tables and graphs.

### Ethical issues

The parents/guardians signed the consent form, and the clinically stable children >6 years of age agreed to participate in the study through the consent form. The study was approved by the Research Ethics Committee of the School of Nursing of the Federal University of Bahia, Brazil (Authorization No. 964.177 and Certificate of Appreciation for Ethical Certification - CAAE 40030314.7.0000.5531) and was registered with the National Commission for Research Ethics. During the collection, the children who presented signs of deterioration, identified by the reference standard, were evaluated and assisted by the on-call staff.

## Results

### Characterization of the sample

Table 1 presents the age groups and the clinical profile of the 271 children evaluated in order to characterize the sample studied. The majority of the children were less than 6 years of age (71.2%), had a clinical diagnosis (87.8%), had no comorbidities (63.1%), and more than half had previously been hospitalized (52.8% %). Of the clinical diagnoses, infections and respiratory disorders were the most prevalent.

**Table 1:** Distribution of the age groups and clinical characteristics of the children evaluated. Feira de Santana, BA, Brazil, 2015

**Clinical characteristics (n = 271)**		**n**	**%**
**Age groups (years)**			
	**6 to 10**	**78**	**28.8**
	**3 to 5**	**56**	**20.7**
	**1 to 2**	**54**	**19.9**
	**<1**	**83**	**30.6**
**Diagnoses**			
	**Clinical**	**238**	**87.8**
	**Surgical**	**33**	**12.2**
**Comorbidities**			
	**Did not present**	**171**	**63.1**
	**Presented**	**100**	**36.9**
**Previous hospitalization history**			
	**No**	**128**	**47.2**
	**Yes**	**143**	**52.8**

### Actual prevalence and prevalence estimated by the test

According to Table 2, the prevalence of clinical deterioration established by the reference standard was 17%. The prevalences found by the BPEWS-Br for scores ≥3 and ≥4 were, respectively, 16.2% and 6.2%. Thus, the prevalence of deterioration found by a score of 3 was the one that was closest to the prevalence obtained by the reference standard.

**Table 2:** Distribution of the prevalences of actual clinical deterioration by the reference standard and that estimated by the BPEWS-Br*, among the children evaluated. Feira de Santana, BA, Brazil, 2015

Actual prevalence and that estimated by the test	n	%
By the reference standard	46	17.0
By the BPEWS-Br*≥3	44	16.2
By the BPEWS-Br*≥4	17	6.2

*Version of the Brighton Pediatric Early Warning Score translated and adapted for the Brazilian context.

### Validity indicators of the BPEWS-Br

Table 3 shows the validity indicators of the BPEWS-Br applied to the population studied for scores ≥3 and ≥4. The higher score produced lower sensitivity and NPV and higher specificity, PPV and likelihood ratios.

**Table 3:** Distribution of validity indicators of the BPEWS-Br*, applied to the children evaluated, according to the scores adopted. Feira de Santana, BA, Brazil, 2015

**Validity Indicators** ^**†**^	BPEWS-Br scores*	
	**≥3**	**≥4**
**Sensitivity**	**73.9 (58.5-85.2)**	**36.9 (23.5-52.5)**
**Specificity**	**95.5 (91.5-97.7)**	**100 (97.9-100)**
**PPV** ^**‡**^	**77.3 (61.7-88.0)**	**100 (77.0-100)**
**NPV** ^**§**^	**94.7 (90.7-97.1)**	**88.5 (83.8-92)**
**LR+**	**16.6 (8.8-31.2)**	**∞** ^**||**^
**LR-**	**0.27 (0.1-0.4)**	**0.63 (0.50-0.78)**

*Version of the Brighton Pediatric Early Warning Score translated and adapted for the Brazilian context.

†The values ​​of the validity indicators were estimated, with 95% CI, by the Wilson method.

**‡**PPV - Positive Predictive Value.

§NPV - Negative Predictive Value.

||Estimate not calculable, divided by “zero”.

### ROC curve

**Figure 1:**
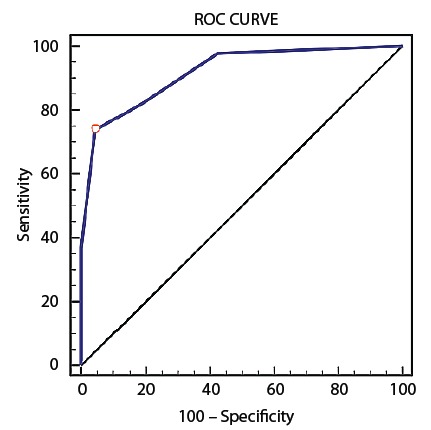
ROC curve* between the BPEWS-Br and the reference standard in the sample studied

According to Figure 1, the BPEWS-Br score of 3 was the most accurate cut-off point for the test, being situated furthest from the 45º line. This means that, in 73.9% of the cases the BPEWS-Br = 3 will be able to detect children with signs of clinical deterioration (true positives), however, this will include 4.5% of children without these signs (false positives).

The area under the ROC curve between the BPEWS-Br and the reference standard was 0.919 (95% CI: 0.973-0.964, *p*<0.001), that is, 91.9% of the times it is used the BPEWS-Br will be able to discriminate the true positives and the true negatives, and will give false results 8.1% of the times.

*Receiver Operating Characteristic Curve -

### Pre-test and post-test probability

Considering the pre-test probability of clinical deterioration of 20%, the probability of positive post-test deterioration (BPEWS-Br ≥3), given LR+ of 16.6, would be 80%. The probability after a negative test (BPEWS-Br <3), given the LR- of 0.27, would be 6%.

## Discussion

The validity indicators obtained in this study showed that, based on the reference standard adopted, the BPEWS-Br proved to be a valid tool, with good performance in the indication of warning signs for clinical deterioration in the children studied, increasing the probability of this event occurring when the score was ≥3.

Some important aspects of the studies that sought to validate the BPEWS in its original, adapted or modified versions need to be analyzed, discussed and compared with data from the present study, such as the various indicators/reference standards for clinical deterioration in children, the cut-off points that indicate the event of deterioration, the validity indicators calculated for the score, the scenarios, the samples and the age groups of the children to whom the score was applied.

Among others, the following reference standards for identification of clinical deterioration have been used to verify the validity of the BPEWS, transfer to the ICU^10^ ^,^ ^12^ ^,^ ^18^ ^,^ ^22^ ^-^ ^23^; call for the RRT; Code Blue - CB (called before cardiorespiratory arrest)^11^; and admission into the hospital^22^ ^,^ ^24^. In this study, none of these standards were used, choosing instead a set of criteria based on the Primary Clinical Evaluation of the Severely Ill Child guided by the AHA and AAP^19^.

The above criteria were followed in order to verify the validity of the BPEWS-Br regarding its actual aim, which is to assist the health team in the early recognition of pediatric clinical deterioration, to provide immediate assistance and to avoid complications arising from late perceived deterioration. This is because, in situations of transfer to the ICU, call for the RRT or CB, the child is likely to be more severe. Admission to the hospital may be motivated by certain situations other than clinical deterioration - for example, for diagnostic investigation or use of medication for the treatment of rare diseases.

From the reference standard adopted, the prevalence of deterioration found in this study was 17%, and the prevalence obtained by the BPEWS-Br ≥3 was 16.2%, values ​​that were very close. In another study in which ICU transfer was used as an indicator of clinical deterioration, it was found that 1.8% of the patients were transferred to the ICU and approximately 24.2% had a score ≥3^10^, values ​​that were very different.

Regarding the cut-off point of the BPEWS, in order to indicate clinical deterioration, some studies considered or found varied scores: 1^12^ ^,^ ^22^, 2^12^ ^,^ ^18^, 2,5^23^, 3^10^ ^-^ ^11^ e 4^11^ ^,^ ^24^. The author of the BPEWS advised that a final score of 4 or a score of 3 in one of the partial components should trigger the call for the RRT, characterizing the clinical deterioration event. However, this behavior could be adapted according to each scenario^6^.

It is necessary to consider that the more the cut-off point is reduced, the greater the sensitivity and the lower the specificity of the score; Thus, healthy patients can be identified as ill by the test (false positives). The ideal is to strike a balance between sensitivity and specificity. In this study, the BPEWS-Br score of 3 was the cut-off point that maximized sensitivity (73.9%) and specificity (95.5%) and obtained the best accuracy (91.9%).

In order to evaluate the performance of the BPEWS, the sensitivity, specificity, predictive values and the areas under the ROC curve were calculated in the majority of the studies^10^ ^-^ ^12^ ^,^ ^18^ ^,^ ^22^ ^-^ ^24^ to obtain the accuracy of the score, with varying results. In some studies, the likelihood ratios^12^ ^,^ ^22^ ^)^ were calculated; The post-test probability, calculated in this study, was not found in any of the studies analyzed.

The likelihood ratio has been an innovative and useful concept in studies of diagnostic accuracy. When multiplied by the pre-test probability, the LR+ and LR- will generate the post-test probabilities, indicating how much the test result will increase or decrease the pre-test probability of a disease^21^, hence its importance.

Thus, the PEWS were not constructed as indicators of emergency situations or of admission to the ICU or the hospital, which imposes certain limits on their use. It is important to note that, depending on the reference standard and cut-off points of the BPEWS, the prevalence of clinical deterioration, as well as performance indicators of the score, may vary and influence the study results.

Regarding the study scenarios, the BPEWS was conceived as an warning instrument for children hospitalized on wards^6^, where urgency and emergency situations are not part of the daily routine of the health team. Therefore, this is a score that can contribute as a support instrument for these teams in the recognition of the clinical severity of the patient. Thus, the majority of the study scenarios for validation of the BPEWS were performed on wards^10^ ^-^ ^11^ ^,^ ^18^ ^,^ ^23^, however, some authors also applied the score in the emergency unit, upon arrival of the patients^12^ ^,^ ^22^ ^,^ ^24^.

For this study, the scenarios used were the clinical-surgical wards and emergency observation/stabilization units, where the patients would already be hospitalized. The emergency units were included as they are places where clinical deterioration is more common when compared to the wards, since, in diagnostic test studies, the spectrum of patients evaluated should be considered, in order to be representative of those who will use the test in the practice^14^.

Regarding the samples studied and the age groups of the children, this study clinically evaluated 271 children from 0 to 10 years of age, trying to standardize the entire evaluation, in order to avoid measurement bias and data loss. Large samples were used in the studies that validated the BPEWS^10^ ^,^ ^12^ ^,^ ^18^ ^,^ ^22^, which may have generated inconsistency in the data collected due to the difficulty of standardization in the evaluations of the patients.

Regarding the age group, other studies^10^ ^-^ ^12^ included patients aged >18 years, however, the BPEWS was constructed for children and adolescents up to the age of 16 years, and its application outside this age group is not recommended. Another important issue is in the evaluation based on primary data, since retrospective studies, based on secondary data, have mentioned the lack of records as a study limitation^11^ ^,^ ^23^.

From what has been discussed, many factors can influence the results of the validation studies of the PEWS, which require caution in their planning and performance. This study validated, for the first time, a PEWS for the Brazilian context, comparing it with criteria of the Primary Clinical Evaluation of the Severely Ill Child, and found encouraging results.

It should be emphasized that a detailed evaluation of the clinical condition of a patient requires careful anamnesis and a physical examination, and it is unlikely that a rapid assessment instrument will be able to fully identify children at risk of deterioration. However, a Pediatric Warning Score (PES), such as the BPEWS-Br validated in this study, can help health professionals improve performance in the early recognition of clinical instability in hospitalized children^13^.

## Conclusion

The results showed that the BPEWS-Br was a valid instrument for the recognition of warning signs of clinical deterioration in the children studied.

The accuracy of the BPEWS-Br is presented in this study, with its reproducibility being shown in a parallel study with 50 children. Multi-center studies should be conducted to expand the evidence for the validity of the BPEWS-Br and to strengthen the arguments for its use in pediatric wards as part of the daily evaluation of hospitalized children in Brazil.
